# Gamma heavy chain disease associated with rheumatoid arthritis: a case report

**DOI:** 10.1186/s13256-021-02696-7

**Published:** 2021-03-17

**Authors:** Gwenvaël Danic, Thomas Dejoie, Hélène Caillon, Aurélie Achille, Pierre Pottier, Christian Agard

**Affiliations:** 1 Internal Medicine Department, Hôtel-Dieu, Nantes University Hospital, University of Nantes, 1 place Alexis Ricordeau, 44093 Nantes, France; 2 Biochemistry Department, Hôtel-Dieu, Nantes University Hospital, University of Nantes, Nantes, France

**Keywords:** γ-Heavy chain, Capillary electrophoresis, Rheumatoid arthritis, Case report

## Abstract

**Background:**

Gamma heavy chain disease (γ-HCD) is a monoclonal gammopathy defined by an abnormal clonal and isolated production of incomplete heavy chain gamma (γ), unable to bind with light chains kappa or lambda. This disease is rare and remains poorly described. Its association to lymphoid neoplasm is well established, but exceptional forms of γ-HCD may also accompany auto-immune diseases. We report here a new case of γ-HCD characterized by an indolent course with a 4-year follow-up, and its association with quiescent rheumatoid arthritis (RA).

**Case presentation:**

We report the case of a 85-year old French white man followed for quiescent anti-CCP+ rheumatoid arthritis treated by prednisolone 4 mg/day and hydroxychloroquine 200 mg/day since 10 years, and a monoclonal gammopathy of undetermined significance for 6 years, who was hospitalized for costal fractures after a fall. Serum protein electrophoresis showed a stable small monoclonal peak, and capillary electrophoresis/immunosubtraction technique identified an isolated clonal γ-heavy chain (HC). Bone marrow aspiration was normal and he had no other lymphoproliferation. The monoclonal peak remained stable after 4 years of follow-up.

**Conclusions:**

In case of monoclonal peak without complete monoclonal Ig on serum protein electrophoresis, the diagnosis of γ-HCD should be discussed and capillary electrophoresis/immune-subtraction is a mean to detect isolated monoclonal heavy chain (HC). Gamma-HC disease is rare, may be associated to RA, and may have an indolent course.

## Introduction

Heavy chain disease (HCD) is a monoclonal gammopathy defined by an abnormal clonal and isolated production of incomplete heavy chain, alpha (α), gamma (γ) or mu (µ), unable to bind with light chains kappa (κ) or lambda (λ) [1]. Gamma-HCD, also known as Franklin’s disease, is rare and remains poorly described [1, 2]. We report here a new case of γ-HCD characterized by an indolent course with a 4-year follow-up, and its association with quiescent rheumatoid arthritis (RA).

## Case presentation

A 85-year old French white man was admitted in 2016 for ischiopubic branch fractures after a scale fall.

His medical past comprised type II diabetes mellitus, steroids-induced osteoporosis, and bacterial pneumonia in 2003 and 2008. He was followed for RA with positive anti-CCP (cyclic citrullinated peptide) antibodies since 1990, that was quiescent since 10 years, with bilateral mild and stable articular erosions of all distal interphalangeal joints. He was also known to have a monoclonal gammopathy of undetermined significance (MGUS) since 6 years, with an unquantifiable peak that had never been analyzed.

His treatment was prednisolone 4 mg/day, hydroxychloroquine 200 mg/day, metformin, alendronic acid, and cholecalciferol.

At admission, he had no fever, neither weight loss or abnormal sweat. Walking was painful because of the fractures, but there were no other bone or articular pain. Joints examination found no signs for arthritis or synovitis. Palpation of liver and spleen was normal and he had no peripheral lymphadenopathies. Physical examination found no other abnormalities.

**Fig. 1:**
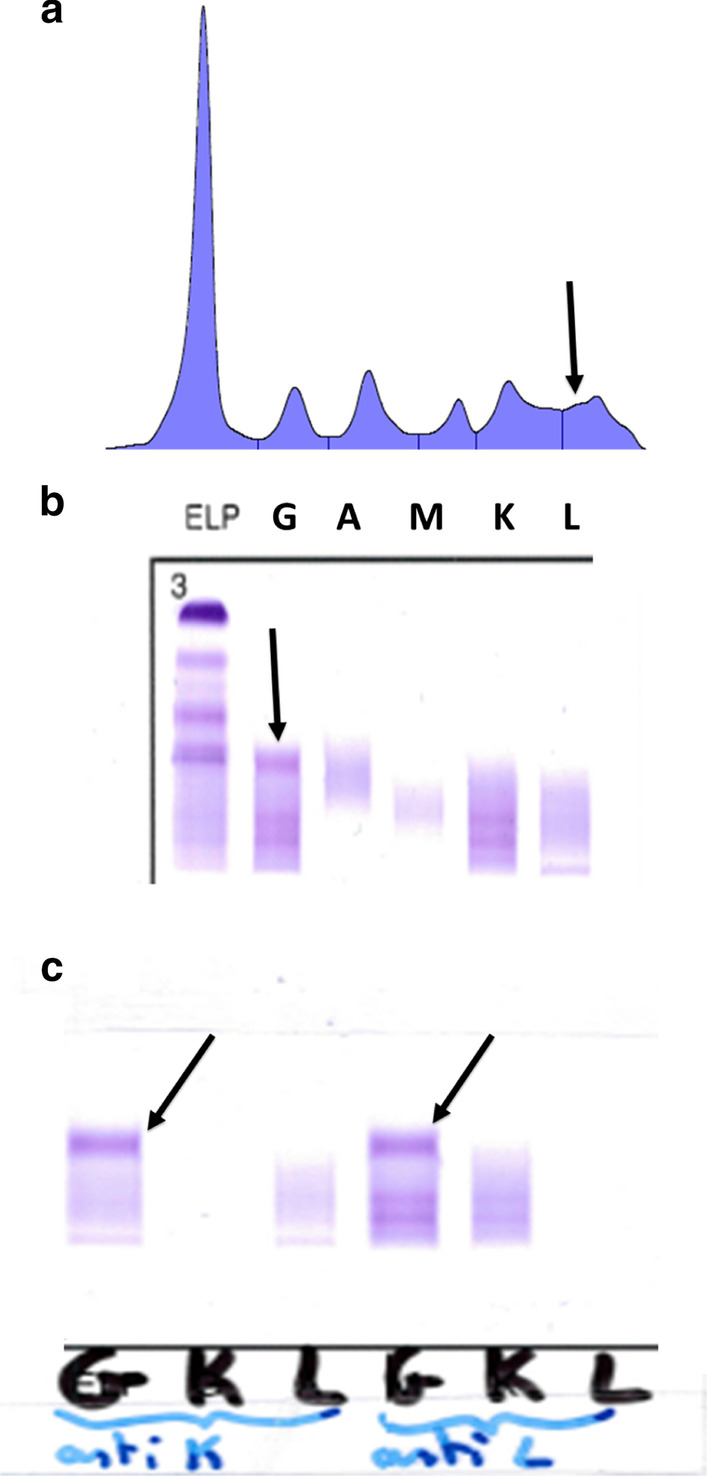
**a** Seric protein electrophoresis showing a small and stable monoclonal peak between beta and gamma zones. **b** Seric protein immunofixation electrophoresis disclosing monoclonal γ-heavy chain (arrow) without any clonal light chain. **c** Protein capillary electrophoresis immuno-subtraction technique showing isolated monoclonal γ-heavy chain. Serum samples of the patient are separately treated with anti-serum anti-κ and anti-λ.

Haemoglobin was normal at 13.3 g/dl, platelets were at 187 G/l, total leucocytes were at 11.61 G/l, with neutrophils at 8.93 G/l, monocytes at 0.91 G/l, and lymphocytes at 1.59 G/l. Blood electrolytes and hepatic parameters were normal, creatininemia was 105 µmol/l, and C-reactive protein was slightly increased at 27 mg/l. A vitamin D deficiency was noted. Immunologic tests found positive rheumatoid factor at 89 UI/ml (normal < 20 UI/ml) and positive anti-CCP antibodies (160 UI/ml, normal < 10 UI/ml). Serum protein electrophoresis found an albumin level at 32 g/l, and there was a beta–gamma block with a possible small monoclonal peak between beta and gamma zones (Fig. 1a). Immunofixation electrophoresis was then performed which found clonal γ-heavy chain without any light chains (Fig. 1b). The highlight of isolated clonal γ-heavy chain was also confirmed using capillary electrophoresis/immuno-subtraction technique (Fig. 1c). G, A, and M immunoglobulins assays were 14.9, 2.4 and 1.96 g/l respectively. Proteinuria was glomerular and non-selective at 1.2 g/day, Bence-Jones proteinuria and immunofixation of urine proteins were negative.

The diagnosis of γ-HCD was made. Thoracic-abdominal-pelvic CT-scan and blood lymphocytes immunophenotyping were normal. We made a bone marrow aspiration and the myelogram was normal, without any dystrophic cells and with 2% normal plasmocytes. Monoclonal peak remained stable after 4 years of follow-up.

## Discussion

In γ-HCD, plasmocytes synthesize and excrete a monoclonal truncated γ-HC which is unable to bind any light chains. On serum electrophoresis, the monoclonal peak may be very small, and even not detectable. The truncated γ-HC preferentially migrates in the beta zone, (mean dosage = 1.59 g/dl) [1, 2], but may migrates in every other zone, depending on its molecular weight which is variable. Capillary electrophoresis/immuno-subtraction is a mean to detect the HC, using anti-κ and anti-λ antiserum, in order to fix normal Ig (immunoglobilin), and then, to look secondary for an isolated HC. Free light chains and/or complete monoclonal Ig may be associated to isolated HC [1, 2]. In most of the cases of HCD, the same HC is also excreted and found in the urine.

Gamma-HCD is a rare disease affecting preferentially females in 65–84% of cases [1, 2]. Clinical complications may be directly related to γ-HCD: skin involvement (cutaneous vasculitis, white atrophy), osteolytic lesions bordered by osteosclerosis, arthritis due to synovial γ-HC deposits mimicking seronegative RA. Gamma-HCD may also affect the kidney, leading to monoclonal Ig deposition disease, nephrotic syndrome, renal failure, and renal HC amyloidosis indeed. Moreover, patients with γ-HCD have a higher susceptibility to pulmonary infections, which was the case in our patient.

Three clusters of γ-HCD patients are identified [1]. In around 60% of cases, γ-HCD is associated to disseminated lymphoma and patients typically have poor condition, weight loss, fever, with enlarged lymph nodes and splenomagly [1–4]. Some of the patients have also palate and uvula edema. Different types of lymphoid neoplasms may be associated to γ-HCD, including chronic lymphoid leukemia, lymphoplasmocytic, marginal zone B cell, follicular, and large B cell lymphomas [1–4]. Some cases of γ-HCD associated to angioimmunoblastic lymphoma, Hodgkin’s disease, multiple myeloma or LGL leukemia have also been described [1–4].

The second cluster affects 25% of patients with γ-HCD, which is associated to localized lymphoma, affecting bone marrow, skin, thyroid, parotid, gastrointestinal or oropharynx tract (MALT lymphoma) [1, 2]. Finally, the third type of γ-HCD is seen in 15% of patients who have autoimmune disease, mainly RA like our patient [5, 6] and rarely systemic lupus erythematosus, Sjöegren’s, vasculitis, autoimmune cytopenia, or myasthenia gravis, without any underlying lymphoid neoplasm [1, 2]. In some rare cases, γ-HCD appears as a MGUS without any other disease. In practice, serum electrophoresis should be made and carefully analyzed in patients with seropositive or seronegative RA, looking for a small peak in the beta zone, in order to investigate for γ-HCD, especially in patients with recurrent pulmonary infections. If γ-HCD is diagnosed, bone marrow analysis is necessary to fully exclude the possibility of underlying plasma cell neoplasm.

The prognosis of γ-HCD is not well-known, depending on underlying co-morbidities. Median survival is around 7 years, with extreme values ranging from 0 to 21. Evolution of γ-HCD can be indolent and slow, which was the case for our patient. Among 15 deaths are reported in the largest published series, 12 were not directly related to γ-HCD or its underlying disease [1]. However, aggressive and poor prognosis lymphoid neoplasms may rapidly occur in some patients [1].

## Conclusion

Gamma-HC disease is rare and may be associated to RA, which were both of indolent course in our patient. In case of monoclonal peak without complete monoclonal Ig on serum protein electrophoresis, the diagnosis of γ-HCD should be discussed and capillary electrophoresis/immune-subtraction is a mean to detect isolated monoclonal heavy chain (HC). Gamma-HC disease should lead clinicians to systematically look for an underlying lymphoid neoplasm.

## Data Availability

Not applicable. Data come from the medical file of the patient.

## Abbreviations

HC: Heavy chain
γ-HCD: Gamma-heavy chain disease
RA: Rheumatoid arthritis
